# Supplementary material for: Presynaptic Rac1 in the hippocampus selectively regulates working memory
Source: bioRxiv. 2024 Mar 18:2024.03.18.585488. Preprint. [Version 1] doi: 10.1101/2024.03.18.585488 (PMC10983896; doi:10.1101/2024.03.18.585488)
Supplement: Supplement 1 [file NIHPP2024.03.18.585488v1-supplement-1.pdf]

bioRxiv preprint doi: https://doi.org/10.1101/2024.03.18.585488; this version posted March 19, 2024. The copyright holder for this preprint (which was not certified by peer review) is the author/funder, who has granted bioRxiv a license to display the preprint in perpetuity. It is made available under aCC-BY 4.0 International license.

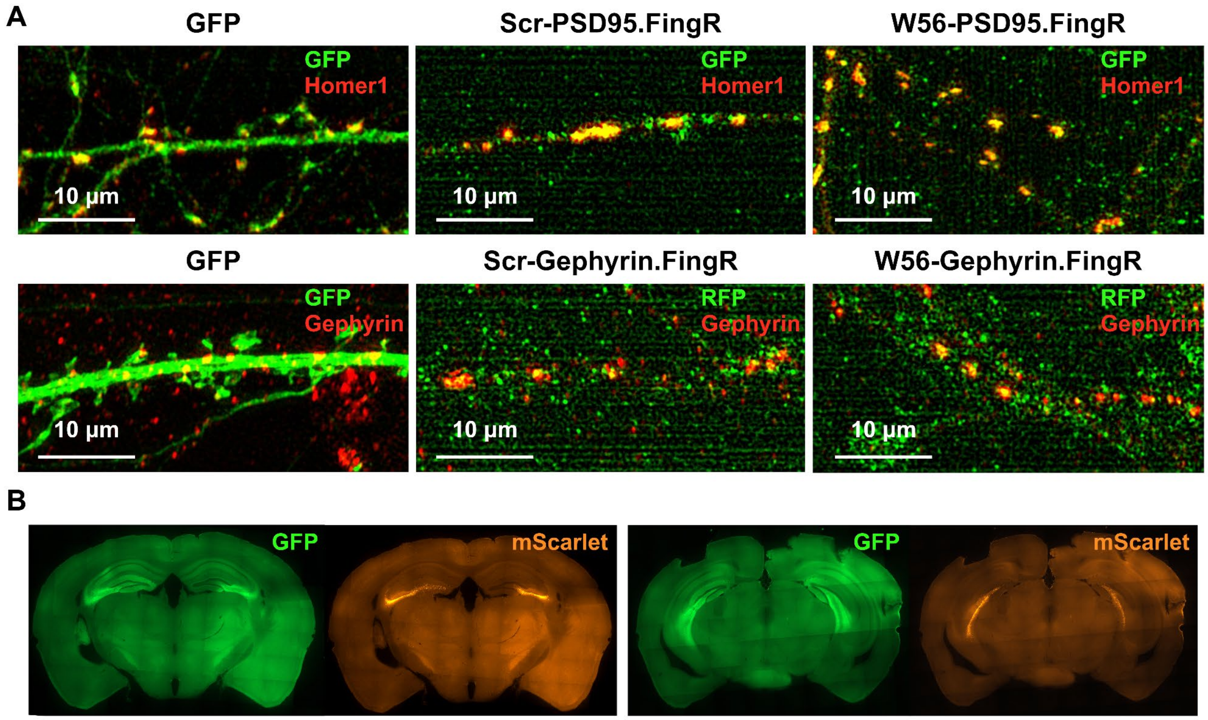

Figure 4-figure supplement 1. Localization of postsynaptic Rac1 inhibitor constructs. (**A**) C57BL/6J primary neuron cultures expressing GFP, the negative control construct, or the postsynaptic Rac1 inhibitor construct. The mScarlet-Gephyrin.FingR constructs were immunostained using an RFP antibody and an Alexa Flour 488 secondary antibody, enabling the imaging of mScarlet in the green channel. (**B**) Representative immunohistochemistry images of brain slices expressing W56-PSD95.FingR-GFP and W56-mScarlet-Gephyrin.FingR. Expression of both constructs was observed in the dorsal (left) and ventral (right) hippocampus.

bioRxiv preprint doi: https://doi.org/10.1101/2024.03.18.585488; this version posted March 19, 2024. The copyright holder for this preprint (which was not certified by peer review) is the author/funder, who has granted bioRxiv a license to display the preprint in perpetuity. It is made available under aCC-BY 4.0 International license.

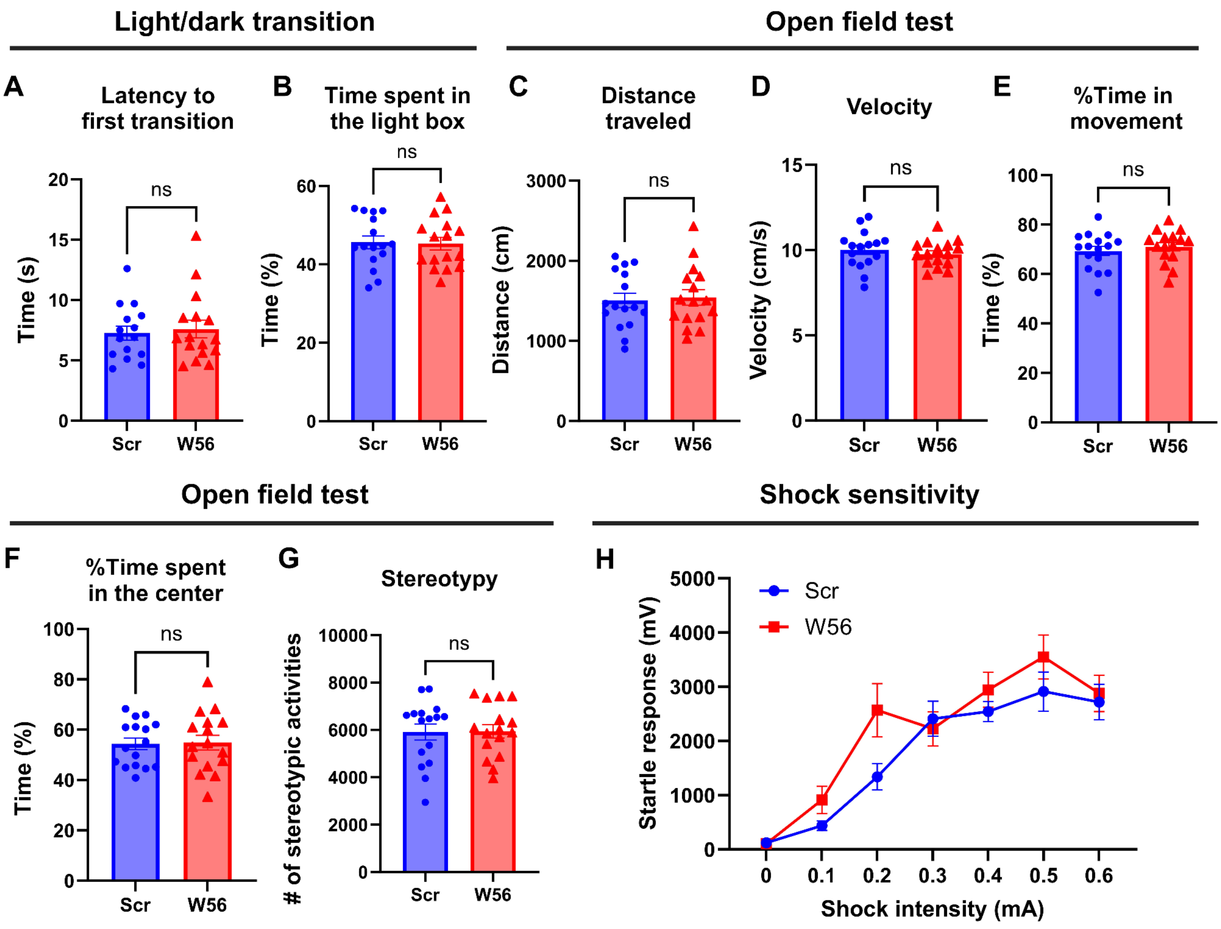

Figure 4-figure supplement 2. Anxiety and general locomotor activity are not affected by postsynaptic Rac1 inhibition. In the light/dark transition, (**A**) latency to the first transition (Scr: 8.350 ± 1.221 s, W56: 7.588 ± 0.7299 s, *P* = 0.9612) and (**B**) time spent in the light box (Scr: 45.60 ± 1.625%, W56: 45.26 ± 1.597%, *P* = 0.8819) were not influenced by postsynaptic Rac1 inhibition. In the open field test, (**C**) distance traveled (Scr: 1,506 ± 89.87 cm, W56: 1,543 ± 95.70 cm, *P* = 0.7775), (**D**) velocity (Scr: 10.02 ± 0.2759 cm/s, W56: 9.768 ± 0.1878 cm/s, *P* = 0.4597), (**E**) percentage of time in movement (Scr: 69.33 ± 1.896 %, W56: 71.07 ± 1.658 %, *P* = 0.4953), (**F**) percentage of time spent in the center (Scr: 54.38 ± 2.265 %, W56: 54.87 ± 2.952 %, *P* = 0.8958), and (**G**) number of stereotypic activities (Scr: 5,914 ± 342.4, W56: 5,946 ± 281.2, *P* = 0.9443) were also unaffected. (**H**) The startle responses elicited by electric shock showed a positive correlation with the intensity of the shock. However, no significant variations were observed across treatment groups (two-way repeated measures ANOVA; Intensity: $F(3.808,110.4) = 32.41$, $P < 0.0001$; Intensity x Treatment: $F(6,174) = 1.313$, $P = 0.2539$). Data are expressed as mean ± SEM with ns. not significant.

bioRxiv preprint doi: https://doi.org/10.1101/2024.03.18.585488; this version posted March 19, 2024. The copyright holder for this preprint (which was not certified by peer review) is the author/funder, who has granted bioRxiv a license to display the preprint in perpetuity. It is made available under aCC-BY 4.0 International license.

from our proteomic data (red) or reported from STRING (blue). (**I**) Phosphorylated peptides that were significantly enriched in the CA proteome. Numbers in gray boxes represent phosphorylation sites. Arrows represent predicted kinase–substrate pairs with high percentile scores (>90). (**J**) Schematic of Syntaxin-1 and Munc18a (PDB ID: 3C98). A red residue represents the phosphorylation site (Ser 109). (**K**) Schematic of Synaptotagmin-1 and SNARE complex (PDB ID: 5CCG). Colors are assigned randomly for different proteins/domains. Data are expressed as mean ± SEM with ** $p < 0.01$.

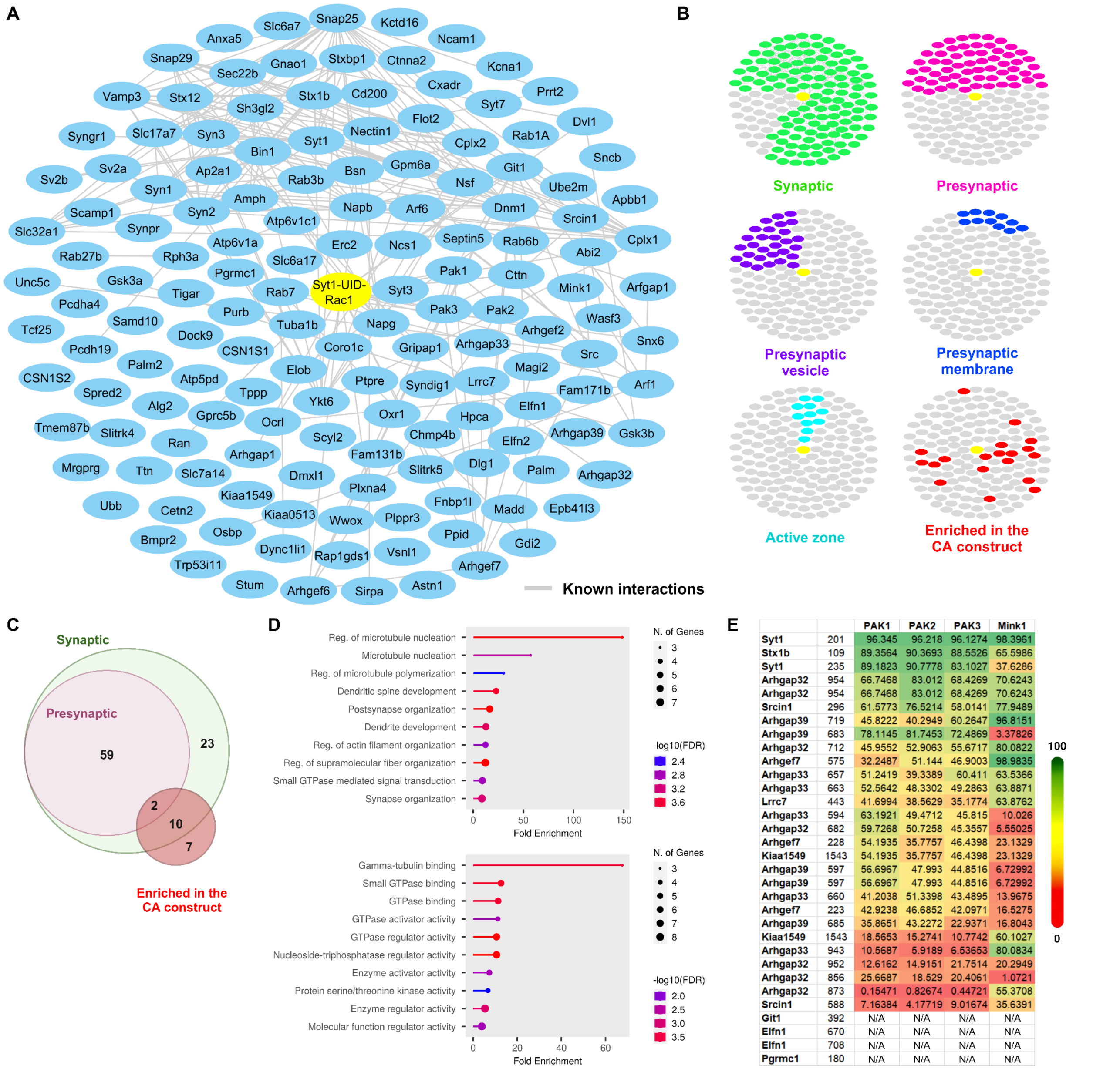

		PAK1	PAK2	PAK3	Mink1
Syt1	201	96.345	96.218	96.1274	98.3961
Stx1b	109	89.3564	90.3693	88.5526	65.5986
Syt1	235	89.1823	90.7778	83.1027	37.6286
Arhgap32	954	66.7468	83.012	68.4269	70.6243
Arhgap32	954	66.7468	83.012	68.4269	70.6243
Srcin1	296	61.5773	76.5214	58.0141	77.9489
Arhgap39	719	45.8222	40.2949	60.2647	96.8151
Arhgap39	683	78.1145	81.7453	72.4869	3.37826
Arhgap32	712	45.9552	52.9063	55.6717	80.0822
Arhgef7	575	32.2487	51.144	46.9003	98.9835
Arhgap33	657	51.2419	39.3389	60.411	63.5366
Arhgap33	663	52.5642	48.3302	49.2863	63.8871
Lrrc7	443	41.6994	38.5629	35.1774	63.8762
Arhgap33	594	63.1921	49.4712	45.815	10.026
Arhgap32	682	59.7268	50.7258	45.3557	5.55025
Arhgef7	228	54.1935	35.7757	46.4398	23.1329
Kiaa1549	1543	54.1935	35.7757	46.4398	23.1329
Arhgap39	597	56.6967	47.993	44.8516	6.72992
Arhgap39	597	56.6967	47.993	44.8516	6.72992
Arhgap33	660	41.2038	51.3398	43.4895	13.9675
Arhgef7	223	42.9238	46.6852	42.0971	16.5275
Arhgap39	685	35.8651	43.2272	22.9371	16.8043
Kiaa1549	1543	18.5653	15.2741	10.7742	60.1027
Arhgap33	943	10.5687	5.9189	6.53653	80.0834
Arhgap32	952	12.6162	14.9151	21.7514	20.2949
Arhgap32	856	25.6687	18.529	20.4061	1.0721
Arhgap32	873	0.15471	0.82674	0.44721	55.3708
Srcin1	588	7.16384	4.17719	9.01674	35.6391
Git1	392	N/A	N/A	N/A	N/A
Elfn1	670	N/A	N/A	N/A	N/A
Elfn1	708	N/A	N/A	N/A	N/A
Pgrmc1	180	N/A	N/A	N/A	N/A

bioRxiv preprint doi: https://doi.org/10.1101/2024.03.18.585488; this version posted March 19, 2024. The copyright holder for this preprint (which was not certified by peer review) is the author/funder, who has granted bioRxiv a license to display the preprint in perpetuity. It is made available under aCC-BY 4.0 International license.

Figure 6-figure supplement 1. Synaptic enrichment and additional analysis of the presynaptic Rac1 CA and DN proteomes. (**A**) Network of biotinylated proteins identified within either CA or DN proteome. Node titles correspond to the gene names. Edges between nodes represent protein-protein interactions previously reported from STRING. (**B**) Clustergrams of proteins that are previously reported as synaptic (green, *n* = 123/149 proteins) or presynaptic (pink, *n* = 61); localized in presynaptic membrane (purple, *n* = 29), presynaptic vesicle (blue, *n* = 11), or active zone (cyan, *n* = 11) as identified through SynGO; are significantly enriched in our Rac1 CA proteome (red, *n* = 19). (**C**) Venn diagram of the biotinylated proteins annotated as synaptic/presynaptic or enriched in the CA proteome. (**D**) GO analysis of the 19 biotinylated proteins enriched in the CA proteome using Biological Process (left) and Molecular Function (right) datasets. (**E**) Table of percentile scores of kinases and specific phosphorylation sites of substrates. The data was retrieved from the Kinase Library.
